# A Co-Adaptive Brain-Computer Interface for End Users with Severe Motor Impairment

**DOI:** 10.1371/journal.pone.0101168

**Published:** 2014-07-11

**Authors:** Josef Faller, Reinhold Scherer, Ursula Costa, Eloy Opisso, Josep Medina, Gernot R. Müller-Putz

**Affiliations:** 1 Institute for Knowledge Discovery, Graz University of Technology, Graz, Austria; 2 Guttmann Institute, Neurorehab. University inst. affil. with the UAB, Barcelona, Spain; 3 Health Science Research Inst. of the “Germans Trias i Pujol” Found., Barcelona, Spain; Interdisciplinary Center (IDC) Herzliya, Israel

## Abstract

Co-adaptive training paradigms for event-related desynchronization (ERD) based brain-computer interfaces (BCI) have proven effective for healthy users. As of yet, it is not clear whether co-adaptive training paradigms can also benefit users with severe motor impairment. The primary goal of our paper was to evaluate a novel cue-guided, co-adaptive BCI training paradigm with severely impaired volunteers. The co-adaptive BCI supports a non-control state, which is an important step toward intuitive, self-paced control. A secondary aim was to have the same participants operate a specifically designed self-paced BCI training paradigm based on the auto-calibrated classifier. The co-adaptive BCI analyzed the electroencephalogram from three bipolar derivations (C3, Cz, and C4) online, while the 22 end users alternately performed right hand movement imagery (MI), left hand MI and relax with eyes open (non-control state). After less than five minutes, the BCI auto-calibrated and proceeded to provide visual feedback for the MI task that could be classified better against the non-control state. The BCI continued to regularly recalibrate. In every calibration step, the system performed trial-based outlier rejection and trained a linear discriminant analysis classifier based on one auto-selected logarithmic band-power feature. In 24 minutes of training, the co-adaptive BCI worked significantly (p = 0.01) better than chance for 18 of 22 end users. The self-paced BCI training paradigm worked significantly (p = 0.01) better than chance in 11 of 20 end users. The presented co-adaptive BCI complements existing approaches in that it supports a non-control state, requires very little setup time, requires no BCI expert and works online based on only two electrodes. The preliminary results from the self-paced BCI paradigm compare favorably to previous studies and the collected data will allow to further improve self-paced BCI systems for disabled users.

## Introduction

Performing specific mental tasks such as movement imagery induces spatio-spectrally specific power decreases (event-related desynchronization, ERD) and increases (event-related synchronization, ERS) in oscillatory bio-electrical activity as measured by the electroencephalogram (EEG) [1], [2]. ERD-based brain-computer interfaces (BCIs) use machine learning techniques to translate patterns of such power changes into control signals [3]. This form of direct communication between brain and environment does not rely on the typical muscular output pathways of the body and can hence serve as assistive technology for individuals with severe motor impairment [4]–[7]. Intuitive, on-demand BCI control, independent of system cues has previously been demonstrated in healthy [8] and disabled users [9], [10] using self-paced BCI systems. For self-paced operation, the BCI ideally detects whether the user is in a state where s/he intends to convey commands (“control state”) or not (“non-control state”). The BCI then triggers commands only in the control state.

ERD-based BCIs can be a promising assistive technology. Their operation, however, is a skillful action that can require a varying amount of training [11]. The typical approach to setup ERD-based BCIs is to first (1) record EEG while the user performs specific mental tasks in a cue-guided paradigm. A BCI expert then (2) trains a statistical classifier based on the collected data. This classifier is then used to (3) provide feedback during an online training session. To attain effective BCI control using a small number of electrodes (e.g. less than 16), it is common to analyze the data from online sessions and to re-train classifiers over multiple sessions. Through this feedback training, the user ideally learns to produce better discriminable patterns of brain activity. This method has been shown to be effective ([4], [5], [7], [9]), but takes time and can be strenuous for the user. Using a high number of electrodes with this conventional training approach can lead to highly effective ERD-based control in only one day of training in able-bodied users ([12]), but is slightly less practical due to the longer setup time.

Co-adaptive ERD-based BCIs on the other side, typically provide feedback for the user's brain-activity as early as possible and continuously adapt the underlying classifier model. In healthy individuals, co-adaptive ERD-based BCIs have proven highly effective both using a low (c.f. [13]–[15]) and a high number of EEG electrodes (c.f. [16]). To a limited extent, co-adaptive ERD-based BCIs have also been shown to be effective for users with severe motor impairment [5], [17], [18].

As of yet, there is no previous work that evaluates the suitability of auto-calibrating and co-adaptive training approaches, to establish ERD-based BCI control for a representative sample of novice users with severe motor impairment. In particular, no previous work in this research direction involved a non-control state which is an important step toward intuitive self-paced operation. Leeb and colleagues ([7]) trained 24 users with motor impairment in a conventional cue-guided paradigm over a maximum of ten sessions, so that half of the users were eventually able to control a spelling application or a tele-presence robot. Among other things, the authors identified auto-calibration and a non-control state especially for self-paced operation as important future research directions. Previous publications about self-paced operation in users with motor impairment were mostly case-studies using conventional, non-automated setup protocols, that required a BCI expert and training over a number of sessions [9], [10], [19].

Our primary aim in this work is to evaluate the effectiveness of a cue-guided, auto-calibrating and online re-calibrating ERD-based BCI training paradigm with a large group of 22 (20 novice) users with severe motor impairment. The BCI requires only six scalp electrodes overlaying the sensorimotor cortex and provides real-time feedback based on only two of these electrodes. The system starts collecting cue-guided mental activity for movement imagery of left and right hand and a non-control class. After approximately five minutes the system auto-calibrates and proceeds to provide visual online feedback for classifying the non-control state against the movement imagery of the particular hand that allowed for higher statistical discriminability. As a secondary aim we want to present preliminary results from a specifically designed self-paced training paradigm that is based on a low-bandwidth user interface adapted from literature [20].

## Methods

### Recording setup

**Figure 1:**
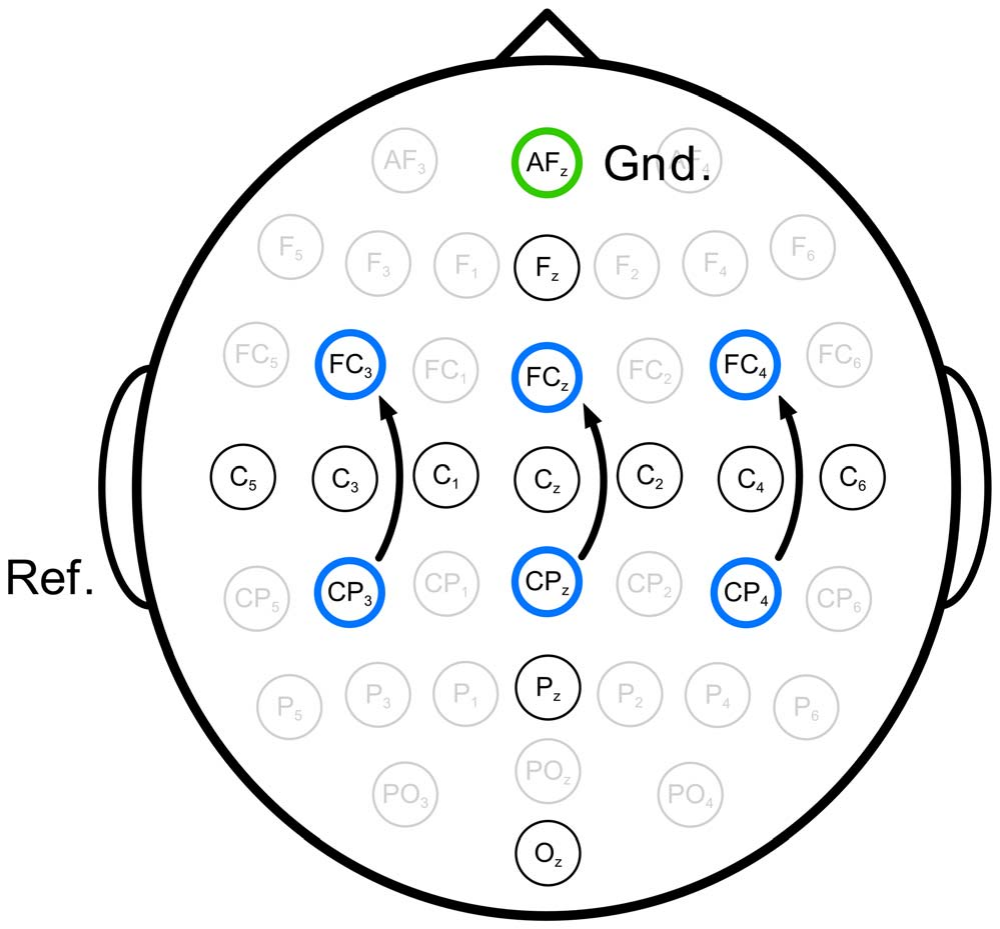
Recorded scalp electrode positions. The three bipolar derivations, indicated by the arrows were considered by the co-adaptive BCI. Feedback was provided from only one of these bipolar derivations. The bipolar derivation selected in the last re-calibration, was also used for the self-paced paradigm. The black circles mark electrodes, recorded for future analyses. The reference electrode was at the left ear-lobe (Ref.) and the ground electrode at AFz (Gnd.).

Six EEG channels were recorded for the BCI. Ten additional channels were recorded for later offline analysis (not presented in this paper). The active electrodes were placed according to the 10/20 System of Electrode Placement (see Figure 1). The signal was sampled at 256 Hz with a band pass filter between 0.5 and 100 Hz and a notch filter at 50 Hz. A biosignal amplifier (g.tec Medical Systems, Graz, Austria) was used for recording.

### Participants

Twentytwo volunteers with severe motor impairment participated in our study (age 37.8 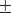 16.0 (SD) years; six female). All participants suffered from motor impairment in all four extremities. The medical conditions were either cervical spinal cord injury (SCI; ASIA A to D according to [21]), polyneuropathy, traumatic brain injury (TBI) or multiple sclerosis (MS). See Table 1 for details. Participant P18 suffered from paralysis of the right eye. All other end users had normal or corrected to normal vision. Participant P17 was in “Locked-in State” according to the definition in [22]. All measurements were conducted at the Institut Guttmann Neurorehabiliation Hospital (Barcelona, Spain). The study, including the measurement protocol and the consent procedure were approved by the local ethics board, “Comitè d'Ètica Assistencial de l'Institut Guttmann”. All participants gave informed, oral consent. In addition, written consent was obtained for every participant. The signed consent forms are stored with the participants' clinical files. In many cases, written consent had to be provided by the participants' legal representatives as many participants were not able to write due to tetraplegia. The participants were instructed in person by caregivers with the support of presentation slides as briefing material.

**Table 1:** Information about the severely impaired participants.

			Hand	Months		
User	Age	Sex	dominance	since injury	Medical Condition	Disability
**P01**	66	F	Right	8	Guillain-Barré syndrome	Tetraparesis
**P02**	21	M	Right	2	SCI C4, ASIA A	Tetraplegia
**P03** ^a^	46	M	Right	24	SCI C4, ASIA A	Tetraplegia
**P04**	19	M	Right	6	SCI C3, ASIA A	Tetraplegia
**P05**	39	M	Right	19	SCI C6, ASIA A	Tetraplegia
**P06**	45	M	Right	3	SCI C7, ASIA C	Tetraplegia
**P07**	60	M	Right	4	Brain Anoxia	Tetraplegia
**P08**	25	M	Right	11	SCI C4, ASIA A	Tetraplegia
**P09**	19	M	Left	5	SCI C4, ASIA B	Tetraplegia
**P10** ^a^	43	F	Right	280	SCI C4, ASIA A	Tetraplegia
**P11**	21	M	Right	6	SCI C5, ASIA B	Tetraplegia
**P12**	65	F	Left	4	SCI C1, ASIA C	Tetraplegia
**P13**	38	M	Right	3	SCI C4, ASIA D	Tetraplegia
**P14**	19	M	Right	66	SCI C4, ASIA A	Tetraplegia
**P15**	47	M	Right	12	SCI C7 and TBI, ASIA A	Tetraplegia
**P16**	42	M	Right	147	SCI C6, ASIA A	Tetraplegia
**P17**	23	M	Right	6	TBI	Locked-in state
**P18**	34	F	Right	74	Multiple Sclerosis	Tetraplegia
**P19**	28	M	Left	5	TBI & brachial plexus injury	Tetraparesis
**P20**	24	F	Right	64	SCI C2, ASIA A	Tetraplegia
**P21**	41	F	Right	9	Hemorrhagic stroke	Tetraplegia
**P22**	66	M	Right	15	Polyneuropathy	Tetraparesis
**Mean**	37.8			35.1		
**SD**	16.0			64.9		

The participants are sorted by co-adaptive BCI performance. The superscript “a” marks the two participants who had used ERD-based BCIs before. TBI stands for traumatic brain injury. Functional scoring for spinal cord injury (SCI) is according to the American Spinal Injury Association (ASIA, [21]).

### Data collection

**Figure 2:**
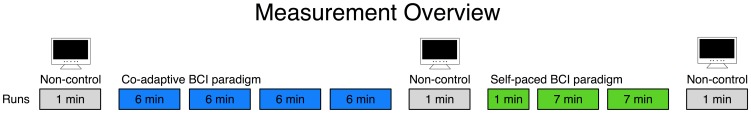
Overview of the measurement procedure. The runs colored in blue were recorded with the co-adaptive paradigm (see Figure 3). The runs colored in green were recorded with the self-paced paradigm (see Figure 4). During the non-control runs, we recorded EEG while participants relaxed with eyes open looking at a black screen. These non-control runs are not analyzed in this paper.

We recorded all EEG data in segments (“runs”). One run lasted one to seven minutes. See Figure 2 for an overview. For the co-adaptive paradigm we collected four runs of data (six minutes per run). There were 36 trials per run and 144 trials total for two classes per participant. For the self-paced paradigm, we recorded three runs of data. The first of these three runs was one minute long and was used to automatically adapt the bias of the classifier. The other two runs were seven minutes long. Two participants (P04 and P10) did not participate in the measurements for the self-paced paradigm.

### Co-adaptive BCI paradigm

**Figure 3:**
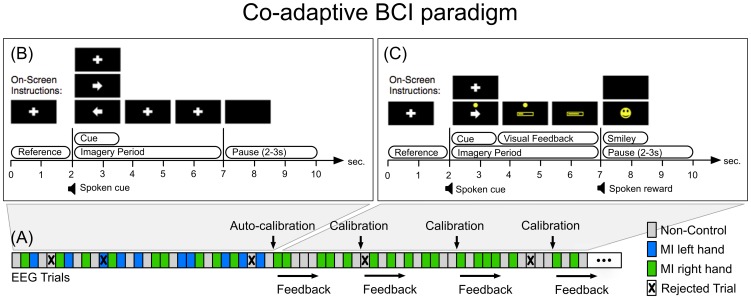
Schematic description of the co-adaptive BCI paradigm. Panel (A) shows how the system initially collected trials for three classes *non-control*, *left* and *right* hand movement imagery (MI left/right hand). Panel (B) shows the trial structure for the “Initial calibration phase”. After nine “artifact-free” trials per class (TPC) were collected the system auto-calibrated, selected one of the hand MI classes and continued to provide visual, real-time feedback. Panel (C) shows the trial structure for the “Online phase”. The system re-calibrated whenever five new artifact-free TPC were available.

The co-adaptive paradigm started collecting data trials for one non-control class and two movement imagery classes (see Figure 3, Panel (A) and (B)). Cues were presented as audio-playback of spoken words and large, well discernible visual shapes, to make the paradigm usable for individuals with visual impairment. Every trial started with a reference period where a white cross was displayed from second zero to two. For this time, participants were instructed to visually fixate the white cross and relax with eyes open.

The visual and audible cue for one of initially three classes was presented at second two. The sequence of cues was random: The class *non-control* was indicated by a white cross and the spoken word “relax”. For this class, participants were instructed to continue to relax with eyes open and to focus on the white cross. For class *left* and *right*, the participants were instructed to sustain kinaesthetic movement imagery (palmar grasp, [23]) of the left or the right hand over the whole imagery period until second seven. The two classes were indicated by a left and right pointing arrow and the audible cues were the spoken words “left” and “right”. No feedback was provided during the “initial calibration phase”. In the background the system continuously identified artifact-congested trials in two steps: First by thresholding amplitude, kurtosis and probability of the band-filtered EEG [24] and second, by identifying trials where at least one feature is an outlier to the distribution of the values for all other trials [15].

As soon as nine artifact-free trials per class (TPC) were available, the system trained one linear discriminant analysis (LDA) classifier for class *left* against class *non-control* and another one for class *right* against class *non-control*. For each classifier, the system chose one of six logarithmic band power features 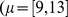 Hz and 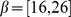 Hz from the bipolars at C3, Cz and C4). The BCI then selected the one MI class with higher cross-validation classification performance against class *non-control* and proceeded to provide continuous, real-time visual feedback only for these two classes for the rest of the measurement (see Figure 3, Panel (C)).

In this “online phase”, the system continued to perform trial-based outlier rejection and re-calibrated the system whenever five new artifact-free TPC were available (see Figure 3, Panel (A)). In every calibration step, the system also trained an autoregressive (AR) filter model (order 11) on all artifact-free trials. See Appendix A for more details on the calibration procedure and how the created classifier model was used in the self-paced BCI training paradigm.

During online operation, the system then applied the inverse transfer function of the AR filter to the EEG and thresholded the residual (prediction error) to detect artifactual activity in real-time [25]. Whenever artifactual EEG was detected, the system displayed a yellow dot (see Figure 3, Panel (C)). The yellow dot, remained on display for 0.5 s after offset of artifact detection. The end users were instructed to try to avoid any activity that would produce EEG artifacts.

To maximize the training effect and motivation in our group of mostly novice users, we only provided positive feedback between second 3.75 and 7 [15], [26], [27]. Specifically, only when the class-label predicted by the LDA matched the true class-label, a yellow feedback bar was displayed within the yellow rectangle seen in Figure 3, Panel (C). The yellow bar extended in length from left to right in proportion to the LDA distance. The users were instructed to try to extend the bar as far as possible. Whenever the predicted class-label did not match the true class-label of the cue, the yellow rectangle stayed empty.

If the predicted class-label and the true class-label matched for longer than a total time of two seconds between second three and seven, the system displayed a smiley and played an audio recording saying “excellent” starting with the pause at second seven. The length of the pause was random between two and three seconds.

### Self-paced BCI paradigm

**Figure 4:**
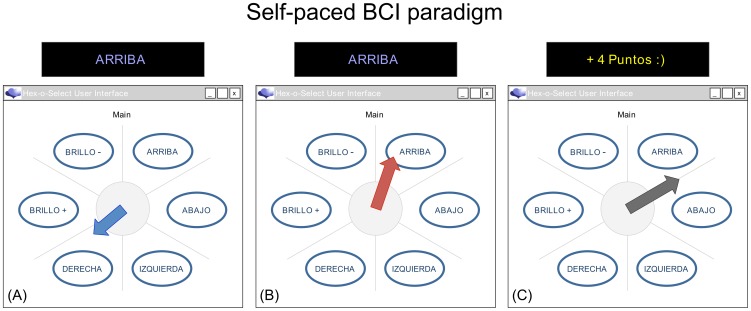
The self-paced BCI paradigm in different states of operation. The head of the arrow was generally rotating clockwise around the center. Panel (A) shows how the arrow is short and colored in blue, whenever class *non-control* is detected. The dialog above the window indicated the next target item. Panel (B) shows how the arrow changed its color to red, when movement imagery was above the activation threshold. The user scored one point for every second the arrow stayed above this threshold in a target segment. Panel (C) shows how the user received feedback if s/he scored at least one point. In this case the arrow stopped rotating and turned grey. After a refractory period of three seconds the paradigm returned back to the initial state depicted in Panel (A).

The self-paced paradigm was based on a validated low-bandwidth input user interface (UI) used in a very similar form in the assistive technology prototype BrainAble [20], [28], [29] (see Figure 4). It typically displays around six menu items in a circular arrangement of segments. An arrow points from the center of the user interface toward one segment at a time. The head of the arrow rotates clockwise around the center so that it takes four seconds to rotate over one segment. The length of the arrow stays at a fixed short length in case the non-control class is detected. The arrow grows proportional to the LDA distance in case movement imagery is detected. When the arrow length exceeds a predefined threshold, the arrow turns red. Keeping the arrow above the threshold for a certain uninterrupted period of time would usually trigger a selection of the menu item in the segment that the arrow is pointing at.

To evaluate the efficacy of this self-paced BCI training paradigm in a reliable and controlled way, we had to instruct the participants as to which menu items to select. We therefore displayed dynamically updated instructions in a dialog box above the UI (see Figure 4). The next target was determined randomly to be two to five segments clockwise after the last target item or the position of the arrow at the beginning of the run. We found this setup to be closest to the real-world case where the user decides autonomously which item to select. The participants were instructed to look at the screen and do nothing, whenever the arrow was pointing to a segment other than the target. For when the arrow was pointing to the target segment, participants were instructed to perform the previously trained movement imagery (either right or left hand).

To improve motivation [15], [26], [27] and to avoid inducing EEG non-stationarities as a result of “perceived loss of controllability” [30], we displayed the actual feedback only when the arrow was pointing to a target segment. When the arrow was pointing at non-target segments we displayed artificially generated feedback, where arrow length varied with gaussian noise around a length below the activation threshold. For target segments, the users always had full control. For every uninterrupted full second users managed to extend the arrow beyond the activation threshold they scored one point (maximum of four possible). If the users scored at least one point, the paradigm stopped for three seconds at the end of the segment and displayed the points in the instruction panel as seen in Figure 4, Panel (C).

### Evaluation

For the co-adaptive paradigm we computed the accuracy for every sample point between second three and seven in the trial and report the peak value. To compare with results in literature, we also computed the Youden index [31] as the difference between true positive and false positive rate at an optimized threshold and dwell-time (range 0.5 to 4 s in steps of 0.5 s). The Youden index ranges from -1 (all targets missed, all non-targets hit) to +1 (all targets hit, all non-targets missed). We identified better than chance performance by comparing to confidence intervals around the theoretical chance level [32]. The threshold level of chance accuracy was 61.0 % (54 TPC; p = 0.01) for the co-adaptive paradigm.

For computing accuracy in the self-paced paradigm we considered true positive (TP), false positive (FP), true negative (TN) and false negative (FN) events. We counted one activation whenever the arrow was continuously extended above threshold for one second. Activations that were triggered while the arrow was pointing at the current target segment were counted as TP. All other activations were counted as FP. Notice, FP activations were not displayed to the user during online operation. If there was no activation throughout a segment, we counted one FN activation in case of a target- and one TN activation in case of a non-target segment. From all segments on average 31.2% were targets, the rest were non-targets. For computing accuracy we corrected the confusion matrices for this class imbalance so that the theoretical chance level was 50%. We conservatively computed the level of statistically significant (p = 0.01) chance accuracy based on the number of target segments for every end user. For statistical comparisons with results from literature we used undirected t-tests for independent samples.

## Results

**Figure 5:**
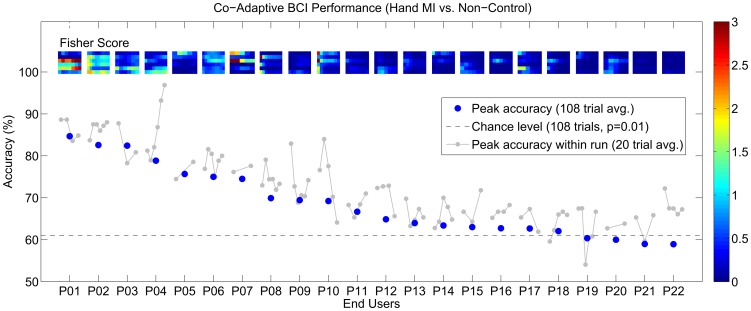
Online performance for all 22 end users. The blue dots show the overall peak accuracy, while the grey dots depict within session performance. The color coded maps show the Fisher criterion [48] over time (left to right) for the features 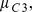 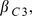 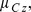 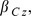 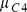 and 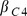 (bottom to top).

**Figure 6:**
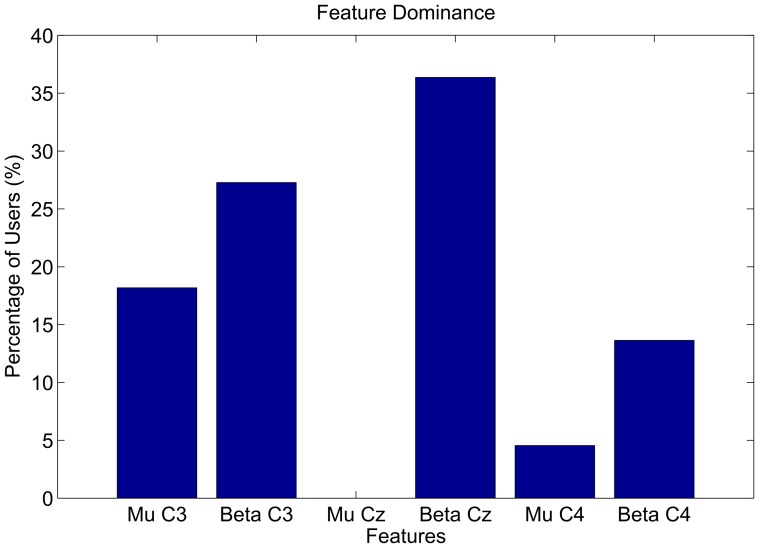
Feature dominance after calibration. Shows for what percentage of users, the different logarithmic band-power features were selected in the final classifier calibration step.

**Figure 7:**
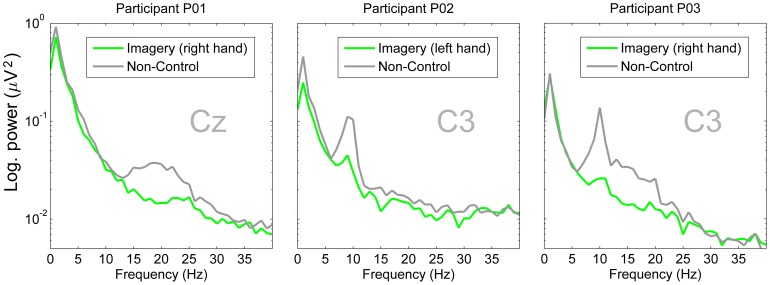
Overview of power spectra. The three panels show power spectra for the three participants for whom the BCI system worked most effectively. For participant P01 and P03, the system selected the *β*-feature and for participant P02 the *α*-feature. Here, all three users control the system by causing oscillatory power of the sensorimotor rhythms to decrease (event-related desynchronization, c.f. [2]).

The co-adaptive paradigm worked with a peak online accuracy of 68.6 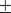 8.2 (SD) %. The performance for 18 of 22 participants was significantly better than chance (p = 0.01). Figure 5 shows the overall peak accuracies as blue dots and the peak accuracies within the session as grey dots. In addition, the figure shows the evolution of feature separability as measured by the Fisher criterion over the recording session for every end user. The system auto-selected the classes *left* and *right* hand movement imagery equally often. From the 50% of end users who scored the highest online accuracy 8 of 11 were using *right* hand movement imagery. Figure 6 shows which features were most dominant in the final calibration step. We found Beta-Cz to be most dominant, followed by Beta-C3, Mu-C3, Beta-C4, Mu-C4 and Mu-Cz. Figure 7 shows exemplary power spectra for the three users, for whom the system worked most effectively. Two end users did not participate in the measurements for the self-paced paradigm. For the other 20 participants, we individually corrected the confusion matrices for class imbalance and found an overall accuracy of 64.4 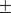 11.0 (SD) %. The accuracies were significantly higher than chance (p = 0.01) in 11 of 20 end users. Table 2 shows detailed results for both paradigms including the accuracies from the corrected confusion matrices for the self-paced paradigm.

**Table 2:** Detailed results for both paradigms.

	Co-adaptive BCI				Self-paced BCI
User	Acc. (%)	Youden index	Selected MI	Feature	Acc. (%)
**P01**	84.7*	0.773	Right	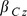	94.2*
**P02**	82.6*	0.715	Left	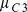	75.6*
**P03** ^a^	82.4*	0.686	Right	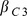	82.1*
**P04**	78.8*	0.573	Right	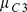	61.8*
**P05**	75.7*	0.373	Left	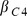	50.2
**P06**	75.0*	0.542	Left	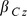	
**P07**	74.5*	0.518	Right	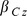	
**P08**	69.9*	0.252	Right	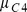	58.5
**P09**	69.4*	0.356	Right^b^	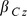	73.4*
**P10** ^a^	69.2*	0.401	Right	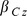	69.8*
**P11**	66.7*	0.218	Right	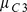	75.1*
**P12**	64.9*	0.227	Left^b^	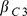	51.8
**P13**	64.0*	0.258	Left	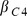	63.4*
**P14**	63.4*	0.268	Right	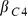	62.9*
**P15**	63.0*	0.120	Right	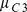	59.4
**P16**	62.7*	0.286	Left	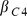	53.3
**P17**	62.7*	0.152	Left	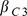	55.3
**P18**	62.0*	0.048	Left	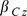	58.2
**P19**	60.4	0.188	Left^b^	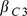	63.3*
**P20**	60.0	0.299	Left	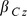	59.2
**P21**	59.0	0.130	Right	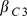	61.2*
**P22**	58.9	0.144	Left	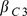	58.8
**Mean**	68.6	0.342			64.4
**SD**	8.2	0.208			11.0

The accuracies for the self-paced paradigm were corrected for class imbalance, so that results are comparable. The superscript “a” marks the two end users who had previously used ERD-based BCIs. The asterisks indicate significantly better than chance (p = 0.01) accuracy. The superscript “b” marks left-handed users. MI stands for motor imagery and Acc. abbreviates accuracy.

## Discussion

### Effectiveness of the cue-guided, co-adaptive paradigm

The co-adaptive paradigm effectively provided better than chance online feedback for the majority (81.8%) of a representative sample of mostly novice severely disabled end users diagnosed with SCI, TBI, polyneuropathy or MS. The system used only two electrodes for online control. At least in healthy users, we previously found that scalp locations with relevant features tend to stay the same between sessions for the same individual [15]. Future training protocols could hence use six electrodes in the first session and mount only the two most relevant electrodes in consecutive sessions. As to feature relevance: Beta features were dominant for most of the users in the final calibration step. Mu features were mostly relevant at position C3; less at the positions C4 and Cz. Features from position C4 were dominant least frequently. We speculate that the factor handedness (19 from 22 users were right handed) might have influenced this outcome. The exemplary spectra for the three most successful users in Figure 7 look as expected, and show how decreases in sensorimotor rhythm power were used to control the BCI systems.

### Comparing to cue-guided, co-adaptive paradigms in healthy users

Vidaurre and colleagues ([13]) presented a highly effective, co-adaptive ERD-based BCI that used 6 electrodes in tests with 12 healthy, novice volunteers. Correcting for statistical chance (p = 0.01) [32] we found our co-adaptive paradigm to work on average 6.7% better than chance (22 end users, none rejected, 20 BCI-novice). The BCI of Vidaurre and colleagues worked on average 11.6% better than chance (12 users, 3 rejected). Even though, this rejection of participants likely skewed the results in favor of the BCI in Vidaurre et al., there is still no significant difference between the results (p = 0.099). This result is highly encouraging, as it indicates that a co-adaptive BCI that supports a non-control state and uses only two electrodes online can work in severely disabled end users with an accuracy comparable to a slightly more complex system in healthy users.

### Comparing to cue-guided paradigms in users with motor impairment

Leeb and colleagues ([7]) validated a conventional ERD-based BCI training protocol with 24 end users (11 with tetraplegia) in a maximum of ten training sessions. The authors discuss, how auto-calibrating and co-adaptive training approaches could expedite BCI setup. Based on their findings, the authors continue to explain how allowing for a non-control state “becomes essential for mentally operating devices over long periods”. Our system implements these thoughtful propositions in that it offers a non-control state, automatically selects the most effective class-combination during auto-calibration and regularly re-calibrates online. The system presented by Leeb and colleagues reached a high Youden index above 0.4 for 41.7% of end users after a maximum of ten training sessions. Our co-adaptive system performed above the same threshold for 31.8% of end users after 24 minutes of training. That means less users reached the same performance threshold with our system in the first session. Still, our system advantageously complements this existing, effective approach, as it offers a non-control state and completely removes the requirement for a BCI expert (even for calibration). After the caregiver mounts the six electrodes and starts the system, users can typically train with real-time feedback based on two electrodes after less than five minutes. Based on literature we would also expect performance of the co-adaptive paradigm to improve over multiple training sessions [5], [13], [15].

### Comparing to cue-guided paradigms in users with SCI

Pfurtscheller and colleagues ([33]) recorded 16 EEG channels from 8 para- and 7 tetraplegic individuals with SCI at lumbar (N_L_ = 1), thoracial (N_Th_ = 7) and cervical (N_C_ = 7) level who were instructed to perform three types of movement imagery in a cue-guided paradigm. Using manual outlier rejection and common spatial patterns (CSP, [34]), the authors found the highest offline classification accuracy between movement imagery of the left hand and both feet. We used these results for comparison. Correcting for statistical chance [32] we found the system in Pfurtscheller et al. to perform 8.2% better than chance (80 TPC; p = 0.01), while our co-adaptive system performed 7.9% better than chance (15 users with SCI; 54 TPC; p = 0.01). We found no significant performance difference (p = 0.943). This result is encouraging as Pfurtscheller and colleagues discuss how their approach was successful with only one of the tetraplegic users. Our co-adaptive system worked better than chance for 14 of 15 tetraplegic end users. Our system classified based on 2 instead of 16 electrodes and automatically provided online feedback after less than five minutes. Our system did further not require manual artifact rejection, feature selection, classifier training or any other interaction of a BCI expert. Most importantly our system supports a non-control state which is important for intuitive, self-paced interaction.

Conradi and colleagues ([35]) calibrated an ERD-based BCI using CSP on 30 minutes of high density EEG (64 electrodes) from 7 BCI-novice individuals with cervical SCI at ASIA levels A or B. The authors found discriminable ERD patterns in four of the participants, computed classifiers and proceeded to record online feedback runs. In the condition “cursor on”, which is most similar to our setup the system worked at 67.7% accuracy (computed as the weighted average of accuracy values in Table 1 in [35]). For our sample of 15 users with SCI (13 BCI-novice; none excluded, ASIA A or B, three with C or D) we found a comparable average online accuracy of 69.9 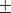 7.4 (SD) %. In comparison, our system does not deliver much higher performance, but our implementation complements the existing, effective approach in other ways: Our system does not require BCI expert interaction and provides online feedback automatically after less than five minutes. The caregiver needs to mount only six electrodes of which only two are used for control, which may be more practical for some applications. Finally, our system offers a non-control state, which is important for self-paced BCI operation.

Rohm and colleagues ([36]) showed how 9 of 10 end users (one rejected due to a classifier problem) with cervical SCI (ASIA A or B) achieved an overall accuracy of 65.7% in a high number of training sessions. While the online accuracy with our co-adaptive system at 69.9 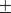 7.4 (SD) % is not much higher, there are some ways how our system complements this existing approach: Instead of more than 13 electrodes, our system requires only six electrodes, from which it only uses two online. Instead of offline training and manual calibration, our system provides feedback automatically after less than five minutes. Most importantly our system supports a non-control state which is important for self-paced operation.

### Effectiveness of the self-paced BCI training paradigm

Several previous case studies ([10], [19], [37]) demonstrated successful self-paced BCI control in individuals with SCI. A recent study showed successful and reasonably flexible control of a spelling application and a tele-presence robot in a large group of users with motor impairment [7]. All of these end users had undergone extensive BCI training typically over multiple sessions and in most cases these systems did not support a non-control state. In our first, simple attempt we found the present self-paced paradigm to work significantly better than chance (p = 0.01) in 11 of 20 end users (majority with SCI; 18 BCI novice). With the exception of P19 and P21, the end users, who achieved better than chance accuracy with the self-paced paradigm had generally also achieved better than chance accuracy previously with the co-adaptive paradigm. Our present approach can complement the effective, existing approaches in that it allows for comparably fast (24 minutes) and fully automatic setup and training without any BCI expert interaction. Typical training protocols to improve performance, like selecting optimal task combinations ([12], [38]–[40]) were performed automatically. Finally, the present self-paced paradigm supports a non-control state and uses only two electrodes during operation.

### Limitations

A limitation of the present setup was that the self-paced paradigm did not work better than chance for as many end users as the co-adaptive paradigm. This was anticipated and can be explained by the fact that in favor of stability we did not yet use fully automatic optimization of the threshold but chose a fixed value for all users. The threshold was fixed to a value which allowed to easily trigger activations with the predefined activation dwell-time of 1 s in the self-paced paradigm. By allowing the users to trigger activations in the target segments, while suppressing erroneous feedback in the non-target segments we were aiming to make this training paradigm more enjoyable and motivating for our mostly novice end users ([27]). In addition we wanted to avoid, that the users' perception of mistakes would introduce additional non-stationarities in the EEG ([30]). Based on the clean data we collected from these end users we can do further analyses and simulations in the future to find system configurations that can automatically optimize threshold, dwell-time and features to allow for more robust self-paced operation.

### Future prospects

In this work we used a co-adaptive BCI paradigm to quickly establish a communication and control channel for users with SCI, TBI, polyneuropathy or MS. The co-adaptive paradigm already supported a non-control state and the generated classifiers worked well in the presented self-paced paradigm. Additional workload measurements in future experiments could help to objectively quantify the merit of supporting a non-control state. Based on the collected data we are working to improve our signal processing methods to attain higher system efficacy. In addition we plan to explore the impact of using non-motor tasks and multi-session training. The present system selected a user-specific control strategy automatically based only on cross-validation accuracy and feature separability. Future implementations could also consider physiological markers in the decision process. In addition to the user population in the present study, future research could also target individuals in minimally conscious state [41]. Finally, co-adaptive BCI training paradigms could also be evaluated for their efficacy as tools in neuro-rehabilitation [42] after neural injuries like stroke [43], [44] or SCI [45], [46].

## Conclusions

We presented a cue-guided, auto-calibrating and online co-adaptive ERD-based BCI training paradigm that allowed for significantly better than chance (p = 0.01) control in 18 of 22 severely disabled users (20 BCI-novice). After only 24 minutes of co-adaptive training, 11 of 20 end users were able to control a self-paced BCI training paradigm with a control proficiency significantly better than chance (p = 0.01). Comparing with literature we found our co-adaptive BCI to well complement existing, effective approaches in that it requires no BCI expert, supports a non-control state and provides feedback based on only two electrodes automatically after less than five minutes.

## Appendix A. Details on classifier calibration and use

For initial class selection, the typical calibration was performed for both class combinations *left* vs *non-control* and *right* vs *non-control* to select the one class combination that showed higher median leave-one-out cross-validation (LooCV) test accuracy. Such choosing of a user-specific task combination had been previously shown to improve ERD-based BCI control proficiency [12], [38]–[40]. The calibration procedure always worked in the following steps on all collected artifact-free trials: First the BCI extracted a total of six logarithmic band-power features (1 second averaging) in the bands 9 to 13 and 16 to 26 Hz ([15], [47]) from bipolar derivations at C3 (FC3 - CP3), Cz (FCz - CPz) and C4 (FC4 - CP4). The system proceeded to select the single feature with maximum discriminability according to the Fisher criterion (cf. [48]) in the classification period from second three to seven within the trial. The BCI then split the classification period into eight adjacent 0.5 s windows and computed LooCV accuracy for every one of theses windows. Specifically the system trained an LDA classifier for the logarithmic band-power values in the 0.5 s time window and then applied the classifier sample-wise to the feature of the whole classification period of the test-trial. Averaging across all test-trials resulted in one accuracy curve of 4 s length for every training window (eight total). The training window, whose LooCV accuracy curve yielded the highest median accuracy over these 4 s was used to finally train the classifier. As a last step the system trained the AR filter model (order 11) of the real-time artifact detection method on all artifact free trials [25]. The system re-calibrated seamlessly in the background whenever five new TPC were available and the most recently trained classifier model was always immediately used in the online system. The last classifier generated in the co-adaptive paradigm was automatically used in the first run of the self-paced paradigm. With an LDA output ranging approximately from -1 to 1, the activation threshold was set statically to 0.5 for all participants. An activation was triggered whenever participants produced above threshold classifier output for a fixed dwell-time of at least 1 s. The system automatically adjusted the bias term of the classifier based on the data recorded in the first run of the self-paced paradigm.
